# Chiral Determination of Naringenin by Ultra-Performance Liquid Chromatography-Tandem Mass Spectrometry and Application in Citrus Peel and Pulp

**DOI:** 10.3389/fnut.2022.906859

**Published:** 2022-06-24

**Authors:** Zhoulin Yao, Shaohui Wu, Hu Zhang, Xianju Feng, Zhen Wang, Mei Lin

**Affiliations:** ^1^Zhejiang Academy of Agricultural Sciences, Hangzhou, China; ^2^Department of Pesticide Science, College of Plant Protection, State and Local Joint Engineering Research Center of Green Pesticide Invention and Application, Nanjing Agricultural University, Nanjing, China

**Keywords:** naringenin, citrus, absolute configuration, chiral analysis, ultra-performance liquid chromatography/tandem mass spectrometry

## Abstract

A chiral separation method of naringenin in citrus pulp and peel was established using ultra-performance liquid chromatography/tandem mass spectrometry (UPLC-MS/MS) in this study. The liquid-phase conditions for separation were Chiralpak IG-3 column at 40°C, mobile phase of methanol, and 0.1% formic acid solution (85/15; v/v). Isovolumetric elution can complete the detection within 5 min. Considering the matrix effect, the matrix standard calibration curve was used for sample quantification. Quantitation was achieved by fitting a calibration curve using a standard matrix. The mean overall recoveries of the two enantiomers from orange pulp were 91.0–110.0% and orange peel were 85.3–110.3%, with relative standard deviations of 1.5–3.8 and 0.9–3.6% at the 0.5, 2.5, 50, and 250 μg/kg levels, respectively. The limit of quantification for all enantiomers in the citrus matrix did not exceed 0.5 μg/kg. Furthermore, the absolute configuration of the naringenin enantiomer was determined by combining experimental and predicted electron circular dichroism spectroscopy, and it was confirmed on a Chiralpak IG-3 column that the first eluting enantiomer was (*S*)-naringenin. The determination of chiral naringenin content in actual citrus samples showed that the naringenin content in hybrid citrus and citrus pulp was significantly higher than that in pomelo. The method established in this study can be used for the determination of naringenin enantiomers in citrus, which is beneficial to variety selection.

## Introduction

Naringenin (2,3-dihydro-5,7-dihydroxy-2-(4-hydroxyphenyl)-4H-1-benzopyran-4-one) is a kind of bitter colorless compound, which is rich in citrus fruit such as grapefruit, orange, lemon, and bergamot. Naringenin has a typical structure of flavanone and contains hydroxyl groups, producing structural analogs for the hydroxyl groups replaced by different functional groups (1).

Naringenin could effectively inhibit the metastasis of prostate cancer (2) and breast cancer (3, 4), and its nano-preparation has potential in preventing and treating cancer (5). It plays a significant role in protecting liver by promoting autophagy (6), mitigates antituberculosis drugs-induced hepatic and renal injury in rats (7), and may be a potential curative therapy for nonalcoholic steatohepatitis (NASH) treatment (8). Naringenin could effectively inhibit the production of proinflammatory cytokines in macrophages (9), alleviate experimental autoimmune encephalomyelitis (10), could be used as a preventive/therapeutic agent for T-cell-mediated autoimmune inflammatory diseases (11), and a potential immunomodulator among therapeutic agents for immune-related diseases (12). Naringenin could reduce insulin resistance in diabetic rats (13) and promote the clearance of metformin (14). It could promote the proliferation of human periodontal ligament stem cells (hPDLSCs) and endothelial differentiation of hPDLSCs (15). Naringenin is a powerful inhibitor of SARS-CoV-2 infection *in vitro* (16), and naringenin-O-carbamate derivatives can be used as multitarget-guiding agents for treating Alzheimer's disease (17). It alleviates cisplatin-induced muscle atrophy by regulating RIPK1/AMPK/NF-κB pathway (18). Naringenin could act against cadmium-induced testicular toxicity in male Sprague-Dawley (SD) rats (19) and combines with hesperetin to treat pancreatic cancer without causing toxicity to normal cells (20). It could alleviate endoplasmic reticulum stress and reduce cell apoptosis (21) and oxidative stress markers of lens in type I diabetic rats (22), which is used for a variety of oxidative stress diseases (23), and widely used in medicine, food, and other fields.

The extraction of naringenin is very important to the analysis results. To some extent, the pretreatment determines the analysis results and directly affects the accuracy of the analysis data. Solvent extraction (24) is a common pretreatment and extraction method for the detection of flavonoids such as naringenin in citrus. However, the extraction solvent is usually volatile, flammable, large in dosage, and toxic. Ultrasonic wave extraction method can promote the wall breaking or deformation of plant cells and tissues, so that the effective components can be extracted more fully. Qiao et al. treated 14 kinds of flavonoids in citrus by ultrasonic sonochemistry (25). Meng et al. extracted flavonoids with ultrasonic-assisted eutectic solvent, which not only reduced the impact on the environment but also improved the extraction efficiency (26).

Previous studies have been done on racemic detection of flavonoid functional components, and newer detection methods mainly include Ultra-high performance liquid chromatography coupled to triple quadrupole mass spectrometry (UHPLC-QqQ-MS/MS) (27), Ultra-performance liquid chromatography/quadrupole time-of-flight mass spectrometry (UPLC-QTOF-MS/MS) (28), and High-performance liquid chromatography-diode array detection (HPLC-DAD) (29). UHPLC-QqQ-MS/MS (24), High-performance liquid chromatography coupled with ultraviolet and electrospray ionization mass spectrometry (HPLC-UV-ESI-MS/MS) (25), Fourier transform infrared spectroscopy, Liquid chromatograph mass spectrometer (LC-MS/MS) (30), and Simplified miniaturized ultrasound-assisted matrix solid-phase dispersion (SM-USA-MSPD-HPLC) (31) have also been used for the detection of racemic naringenin, all of which can meet the experimental needs.

**Figure 1:**
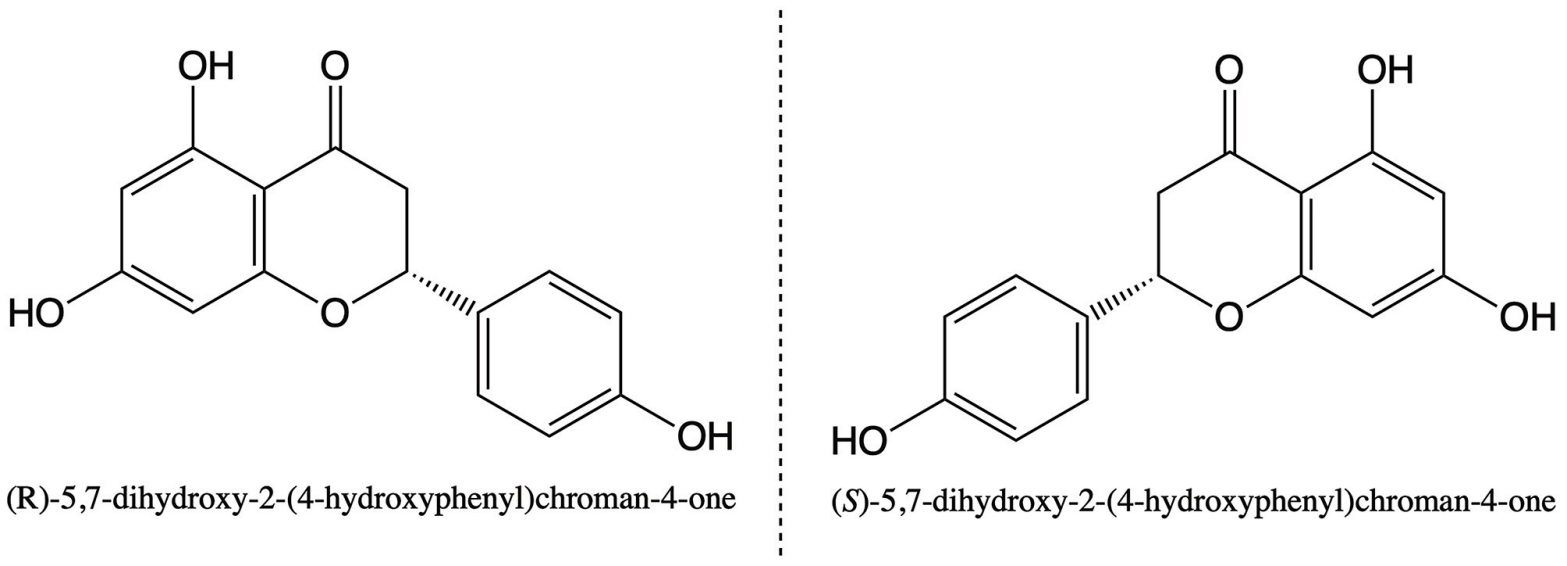
Chemical structures of naringenin enantiomers.

Naringenin has a single chiral center at the C-2 position, which is responsible for its enantiomeric properties (Figure 1). Enantiomers have nearly identical physical properties and differences in enantiomers become apparent in their interactions with other chiral molecules, such as enzymes. The interaction between chiral naringenin and chiral substances is likely to produce different products, especially many biochemical reactions closely related to organisms are related to the chirality of substances. But there is still no research on the absolute configuration and functional studies of chiral naringin monomer. This is a significant research field. When the biological activities of two enantiomers are opposite, the study of chiral components is particularly important.

The instrumental resolution methods of chiral naringenin are mainly the combination of HPLC and HPLC-MS. At present, the research progress is only in the resolution of enantiomers, and the absolute configurations of naringenin *R*- and *S*- have not been determined. As a complete detection method, they all need to be improved. Gaggeri *et al*. separated several kinds of fruit juice naringenin by high-performance liquid chromatography and finally confirmed that the chiral column Chiralpak AD-H was eluted with pure methanol (without acid or alkaline additives), and the separation was high, but the separation time was long, and the absolute configuration and methodology were not confirmed (32). Si-Ahmed et al. separated naringenin by phenyl carbamate-propyl-β-cyclodextrin chiral stationary phase *via* nano-liquid chromatography. This method only ensured the separation of two monomers and also did not confirm the absolute configuration and methodology (33). High-speed counter current chromatography developed by Wang et al., combined with copper (II)-chiral ionic liquid complex and hydroxypropyl-cyclodextrin, focused on the separation of the two monomers; the absolute configuration and methodology were not confirmed, the separation time was long, and the mobile phase was complex (34). Barawska et al. published two studies on the chiral separation of naringenin which were similar in content, and naringenin isomer was separated using the Reversed phase-ultra-high performance liquid chromatography coupled to diode array detection (RP-UHPLC-DAD) (35, 36). However, mass spectrometry cannot confirm the three-dimensional structure but can only determine the quality. Therefore, it is necessary to establish a perfect resolution method for chiral naringenin.

In this article, a complete enantioselective separation method for naringenin in citrus peel and pulp was determined by ultra-performance liquid chromatography/tandem mass spectrometry (UPLC-MS/MS). The qualitative and quantitative ion pairs for the determination of naringenin were optimized and determined. The liquid-phase conditions for the resolution of chiral naringenin were determined by selecting chiral chromatographic columns and optimizing the system of reversed-phase flow conditions. The analytical methods of naringenin enantiomers in citrus peel and pulp were evaluated from the aspects of linearity, recovery, matrix effect, and limit of quantification (LOQ). The absolute configuration of the naringenin enantiomer and the elution order on the chiral column were determined by comparing the experimental electronic circular dichroism (ECD) spectrum with the predicted. The reliability of the chiral analysis method was verified by the determination of naringenin enantiomer in actual citrus samples. The establishment of this method supports further use of molecular biology techniques to study the gene regulation mechanism and metabolomics of citrus chiral naringenin, and provides technical support for improving the development and utilization of high-value citrus resources.

## Materials and Methods

### Experimental Materials

The racemic naringenin standard (purity 99.7%) was purchased from ANPEL (Shanghai, China). Naringenin stock standard (approximately 1,000 mg/L) was obtained by dissolving in HPLC-grade methanol. Enantiomers of naringenin with optical purity ≥ 98.0% were prepared by Shanghai Chiralway Biotech Co., Ltd. (Shanghai, China). Each enantiomer of naringenin stock standards (approximately 1,000 mg/L) was prepared in HPLC-grade methanol. The standard solutions of naringenin enantiomer (150 mg/L) were used to measure the experimental ECD spectrum. All standard solutions were stored in the refrigerator at −20°C in the dark.

Formic acid (≥96% purity) was bought from TEDIA (Fairfield, USA). HPLC-grade methanol and acetonitrile were bought from Merck (Darmstadt, Germany). Ultra-pure water was prepared using the Milli-Q water purification system (Millipore Corporation, Billerica, USA). All other chemicals used in this study were obtained from commercial sources.

### Instruments

UPLC-MS/MS analysis was performed on Sciex 5500+ triple quadrupole mass spectrometer equipped with an ESI source and an Exionlc ad system ultra-high performance liquid chromatography (AB SCIEX, USA). The system was controlled and the data were collected using the SCIEX Analyst OS software package (version 1.7.1). The data were analyzed using the SCIEX OS software package (version 2.0.0.45330).

Chromatographic separation of naringenin was performed on six chiral analytical columns. Cellulose tris (3,5-dimethylphenylcarbamate) (Lux Cellulose-1), cellulose tris (3-chloro-4-methylphenylcarbamate) (Lux Cellulose-2), cellulose tris (4-methylbenzoate) (Lux Cellulose-3), and cellulose tris (4-chloro-3-methylphenylcarbamate) (Lux Cellulose-4) were purchased from Phenomenex (Torrance, USA). Amylose-tris (3-chloro-5-methylphenyl carbamate) (Chiralpak IG-3) was purchased from Daicel (Tokyo, Japan). All five columns were sized 150 mm × 2.0 mm i.d. packed with 3-μm particles. Hydroxypropyl-β-cyclodextrin (InfinityLab Poroshell 120 Chiral-CD) was purchased from Agilent (CA, USA), and the column was sized 150 mm × 2.1 mm i. d. packed with 2.7-μm particles.

### Methods

#### Sample Preparation

The attachments on the surface of citrus samples were gently wiped off, diagonal parts were taken by diagonal division method, peel and pulp were separated, chopped, and mixed well, then they were put into a food processor and thoroughly smashed so that the samples can be tested, and finally they were put inside sub-packaging containers for later use. The pulp of wide peel orange and sweet orange keeps capsule coat, and the pulp of pomelo, seed, and capsule coat were removed. All samples were kept at −18°C until they were analyzed, which were held in zipper-top bags to prevent humidity changes until analysis.

#### Treatment of Citrus Pulp and Peel

**Figure 2:**
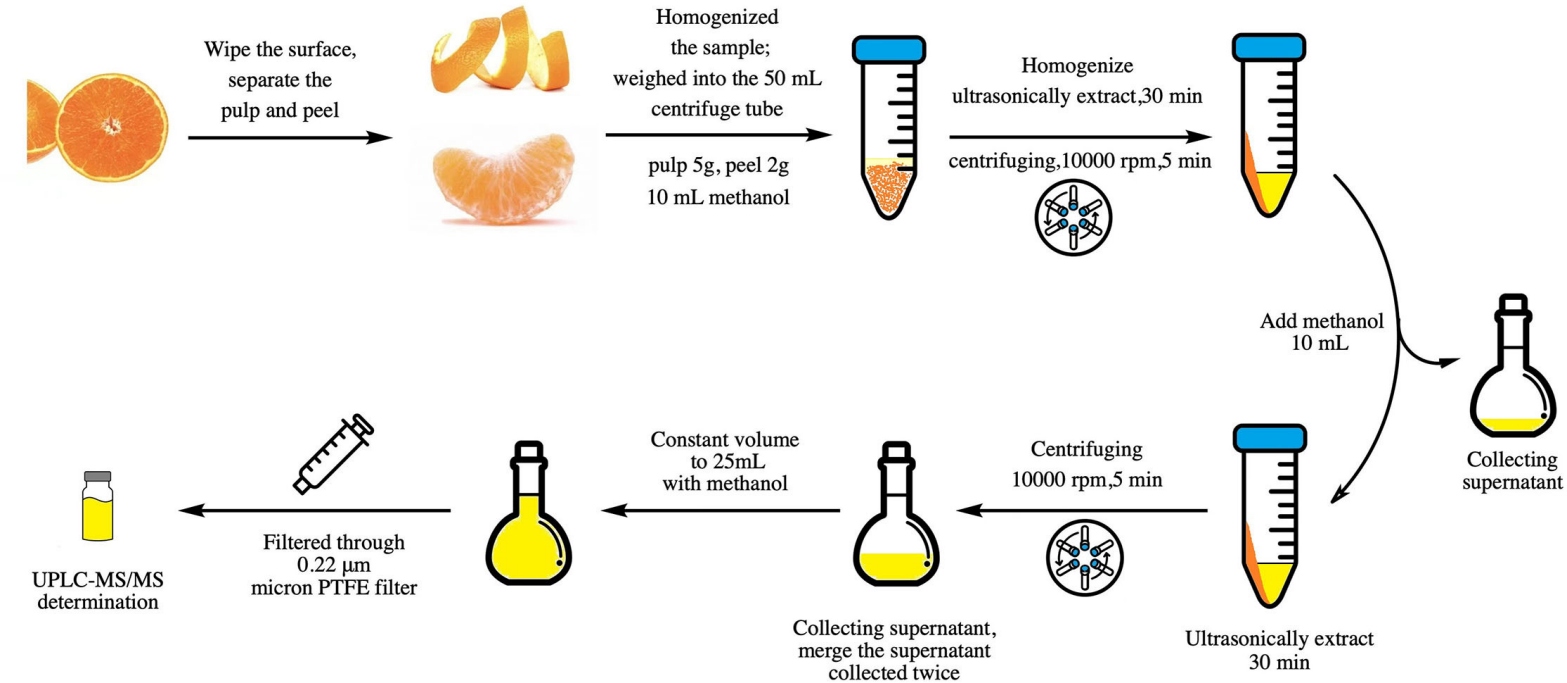
Schematic diagram from sample preparation to sample pretreatment.

A total of 5 g of homogenized orange pulp or 2 g of homogenized orange peel was weighed in a 50-ml polypropylene centrifuge tube. Then, 10 ml methanol was added, homogenized, ultrasonically extracted for 30 min, and centrifuged at 10,000 rpm for 5 min, and the supernatant was collected. Then, 10-ml methanol was added to the residue, the ultrasonic extraction again was repeated, and the supernatants were combined and extracted twice. Finally, the volume of the two extracts was fixed to 25 ml with methanol and then mixed evenly. The solution was put inside a syringe, filtered through 0.22-μm micron PTFE filter, and put inside injection vials for UPLC-MS/MS determination (Figure 2).

#### UPLC Conditions

The mobile phase was methanol or acetonitrile with water (containing 0.1% formic acid solution). The flow rate was set to 0.3 ml/min. The injection volume was 2 μl. All solutions were required to pass through 0.22-μm Teflon filter membrane (ANPEL, Shanghai, China) for use.

#### ECD Conditions

The CD spectrum of the naringenin enantiomer was determined using a Jasco-J815 circular dichroism spectropolarimeter (serial no. A010961168). Spectral data were collected at a scanning speed of 100 nm/min over the wavelength range of 180–400 nm. The data pitch was 1 nm and the bandwidth was 2.00 nm. The average of the three scans was used as the final data.

## Results and Discussion

### MS Conditions

**Figure 3:**
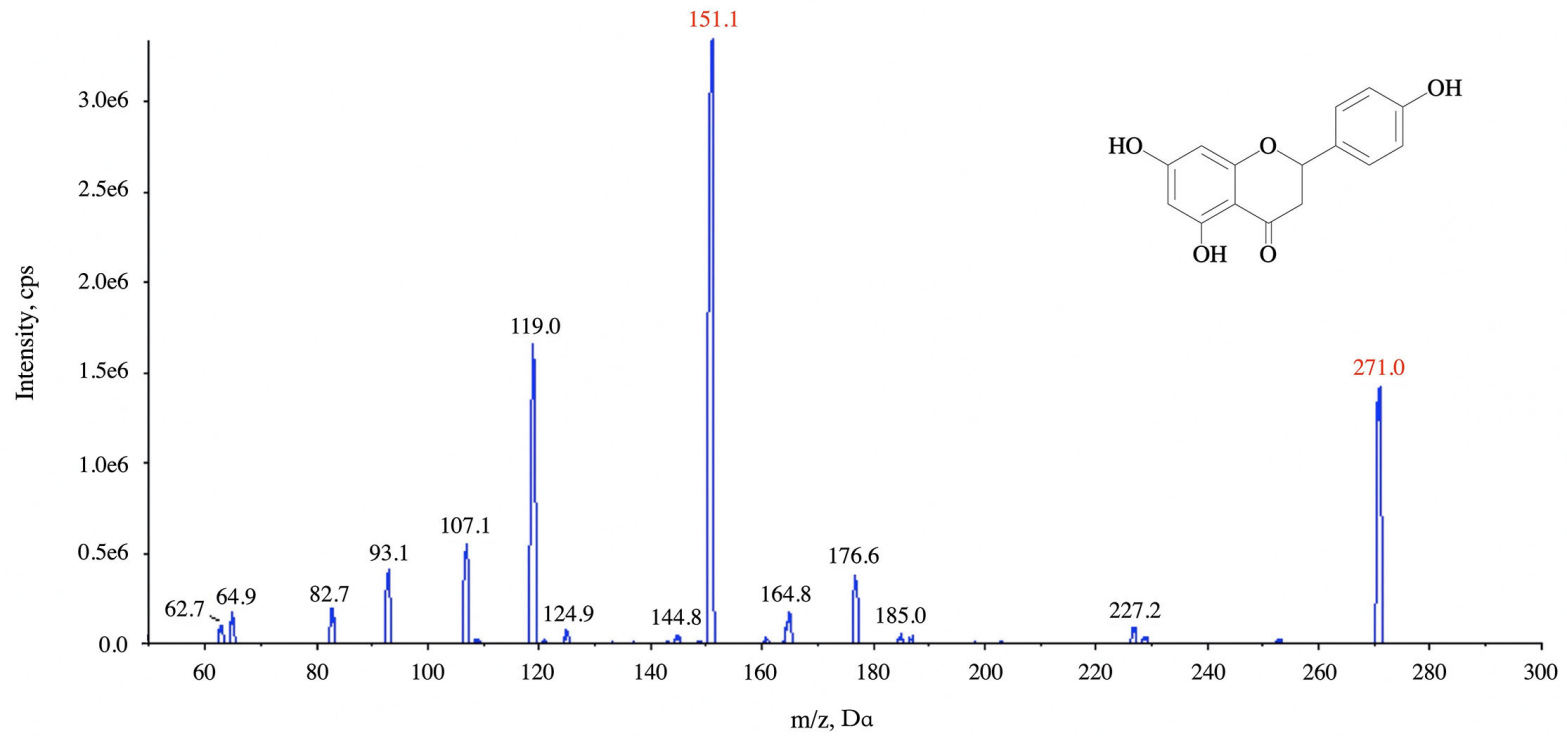
Tandem mass spectra of ion at *m/z* 151.1 and *m/z* 119.0 with the collision energy at −26 and −35 eV, respectively.

The ion optimization conditions of naringin are shown in Figure 3, and the anion mode of selective reaction monitoring was finally determined. The ESI (-) source conditions were as follows: ion spray voltage −4,500 V, spray needle temperature 550°C, collision gas 9 U, and curtain gas 35 psi. The collision energies were −35 and −26 eV, respectively, while the dwell time and declustering potential were 10 ms and −100 eV. For naringenin, the transition *m/z* 271.0 > 119.0 and *m/z* 271.0 > 151.1 were used for quantification and confirmation, respectively.

### The Absolute Configuration of Naringenin Enantiomers

**Figure 4:**
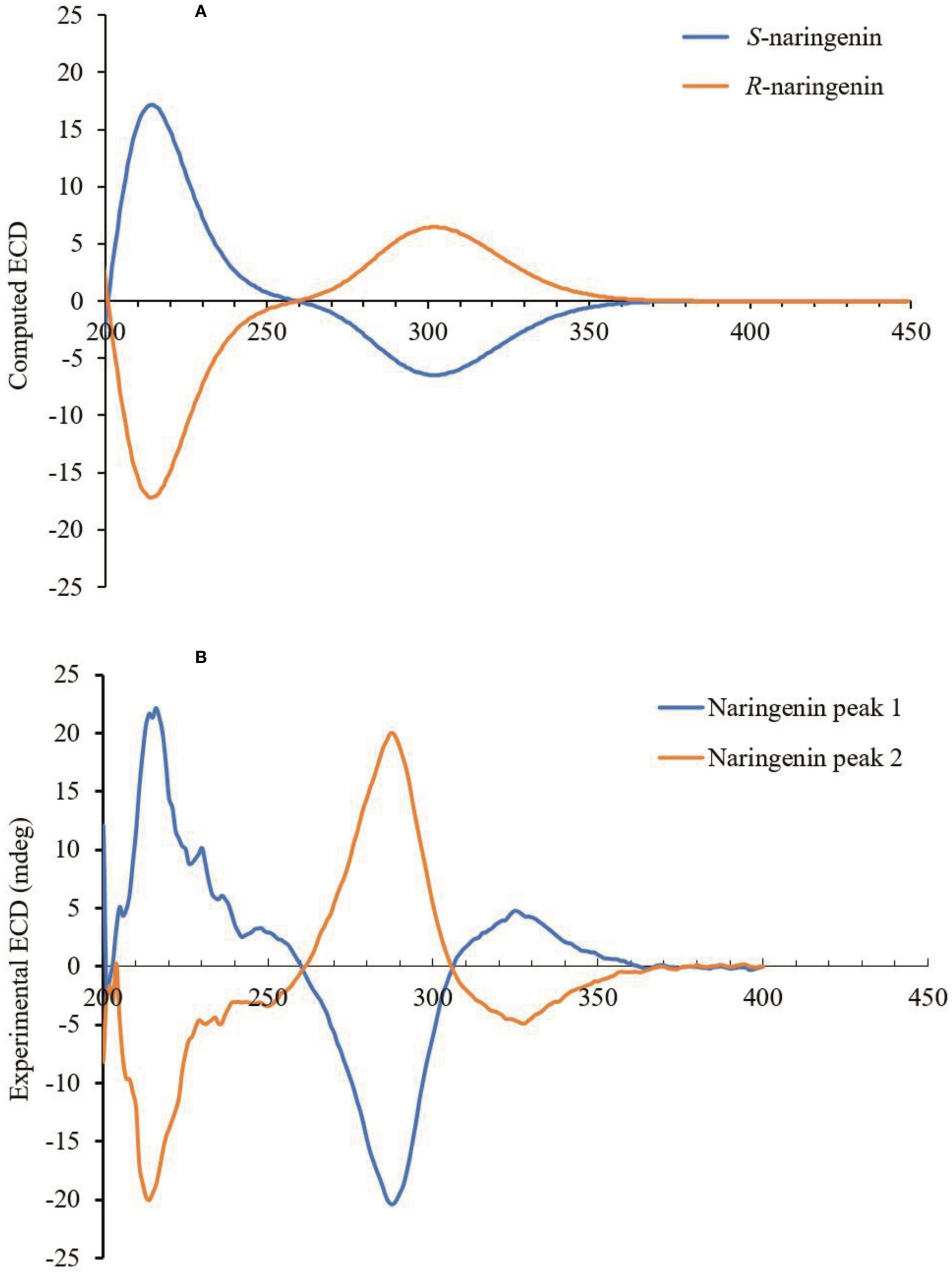
**(A)** Predicted ECD spectra of naringenin enantiomers. **(B)** Experimentally measured ECD spectra of naringenin enantiomers in acetonitrile (150 mg/L).

The experimental and computational ECD spectrum fitting technology was used to determine the absolute configuration of the chiral naringenin molecule in this study. The CD spectrum of a pair of enantiomers is mirrored about the CD = 0 axis. Figure 4B shows the CD spectrum of the naringenin enantiomer in methanol. The CD spectra of naringenin enantiomers were mirror images of each other, and the absorption intensity varied with wavelength. Maximum absorption occurs at 221 nm with no or only weak CD absorption above 370 nm. The wavelength in the range of 200–370 nm was suitable for enantiomeric identification of naringenin from the chromatogram.

We applied the time-dependent density functional theory (TDDFT) method to predict the ECD spectra of naringenin in methanol. The conformational analysis of each naringenin enantiomer was carried out using the Conformational Search module of the ComputeVOA (BioTools Inc., Jupiter, FL), using the MMFF94 force field, and predicting 12 of each enantiomer conformers with the energy of 3 kcal/mol. The stable conformation of two enantiomers was optimized through a molecular mechanics force field (MMFF94), and the lowest energy conformers of each enantiomer were filtered using density functional theory (DFT) at the B3LYP/6-31 + G^*^ level in methanol. These 12 structures were optimized and CD was calculated by TDDFT method at the B3LYP/6-31+G^*^ level in the Gaussian 09 package. The individual spectra obtained were added according to Boltzmann statistics, which resulted in the predicted overall CD curve. Finally, the AC of the naringenin enantiomer was determined by comparing the sign of the experimental ECD with the calculated spectrum. Overall curves of calculated ECD (Figure 4A) obtained by TDDFT calculation and experimental ECD (Figure 4B) showed good agreement. We were able to correctly determine the configuration of the enantiomers of naringenin, and peaks 1 and 2 were identified as *S*-naringenin and *R*-naringenin, respectively.

### Effect of Column and Mobile Phase

The effects of six different chiral stationary phases on the enantiomeric separation of naringenin were investigated systematically. The flow rate was 0.3 ml/min, and the column temperature was 40°C. The mobile phase was initially set to 95% methanol or acetonitrile with 0.1% formic acid in water, and the ratio of the mobile phase was continually adjusted based on the separation. The organic phase ratio was not <30%, depending on the column characteristics. With the increase in the content of aqueous solution, the separation effect will be enhanced. However, high water content increases the retention time and the peak width.

**Figure 5:**
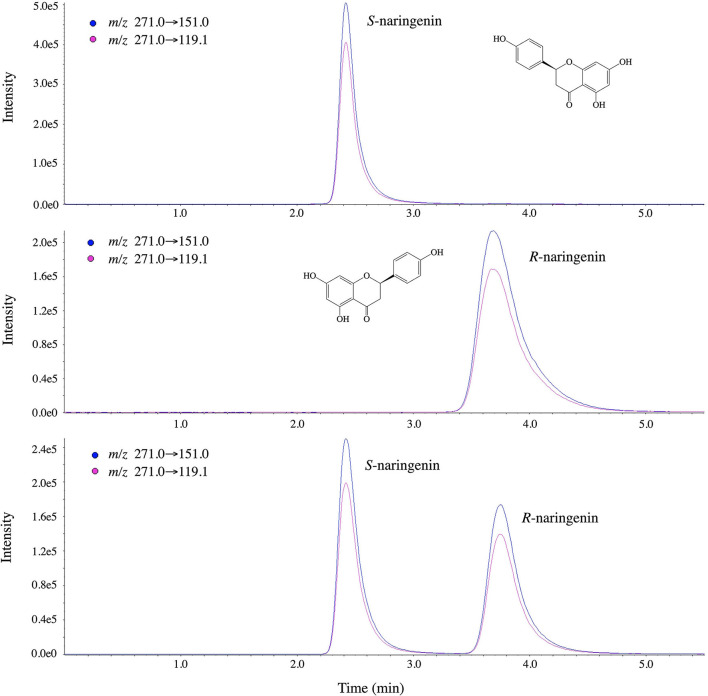
Chromatograms of outflow order of naringenin on IG-3 chiral column.

A better resolution and more sensitivity enantioselective separation of naringenin occurred on Chiralpak IG-3 in the solvent system of methanol containing 0.1% formic acid solution (85/15; v/v) at the column temperature of 40°C. Under the optimized condition, a baseline separation of the enantiomers of naringenin was achieved in <5 min. The outflow order of racemic naringenin under this method was determined by injection of naringenin standard solution, while the chromatograms are shown in Figure 5, in which (*S*)-naringenin flows out first, followed by (*R*)-naringenin.

### Method Validation

#### Linearity and Sensitivity

Excellent linearities were observed for the enantiomers (*R*^2^ ≥ 0.9987), which were adequate for enantiomeric-specific quantitative trace analysis. Matrix effect (*M*_e_) would affect the quantitative analysis results of target compounds. In this study, the parameter *M*_e_ was used to evaluate the matrix effect of naringin on different citrus substrates. *M*_*e*_ = (*k*_*m*_/*k*_*s*_ −1) × 100, where *k*_*m*_ is the slope of matrix standard curve and *k*_*s*_ is the slope of solvent standard curve. When *M*_e_ > 10%, it indicates that there was obvious matrix enhancement effect. When *M*_e_ < −10%, it indicates that there was obvious matrix inhibition effect. −10% ≤ *M*_e_ ≤ 10% indicates that the matrix effect is not significant (37). *S*-naringenin and *R*-naringenin in pulp sample had obvious strong interference and matrix effect as shown in Table 1, which may be related to the fact that the pulp matrix contains a lot of water, which was beneficial to ionization in mass spectrometry. Matrix-matched calibration standards were utilized to eliminate the matrix effect and obtain more accurate determination results in samples in this study.

**Table 1:** Comparison of solvent and matrix standard curves and matrix effects of naringenin enantiomers.

**Enantiomers**	**Matrices**	**Calibration curve**	**Correlation coefficient**	**Matrix effect**
			***(R*** **^2^)**	**(%)**
*S*-naringenin	methanol	*y* = 10,440*x* – 41,712	0.9,992	/
	orange pulp	*y* = 14,883*x* + 54,434	0.9,990	42.6
	orange peel	*y* = 10,079*x* – 13,455	0.9,992	−3.5
*R*–naringenin	methanol	*y* = 11,065*x* + 21,490	0.9,987	/
	orange pulp	*y* = 12,543*x* – 25,435	0.9,992	13.4
	orange peel	*y* = 11,130*x* – 47,841	0.9,985	0.6

#### Recovery and Precision

Naringenin was spiked and recovered in citrus matrix. According to the above pretreatment method, it was injected into the sample for detection under the same instrument conditions. The concentrations of each enantiomer in citrus pulp and peel were 0.5, 2.5, 50, and 250 μg/kg, respectively, with five replicates for each addition. The samples were left for 1 h to ensure that the spiked naringenin was evenly distributed. The added recovered samples were analyzed and the recovery was calculated by comparing the measured concentration to the added concentration. The chiral naringenin content in the added samples was subtracted from the blank matrix sample. The analytical precision and accuracy were evaluated by reproducibility studies (Table 2) and expressed by relative standard deviation (RSD) to validate the chiral naringenin using the UPLC-MS/MS method. The recovery of the method was 85.3–111.2%, and the RSD was 0.9–3.8%. It presented satisfactory mean recoveries and precision for both naringenin enantiomers.

**Table 2:** Recoveries of naringenin enantiomers in orange matrix (*n* = 5).

**Enantiomers**		**Fortified level/**		**Fortified level/**		**Fortified level/**		**Fortified level/**	
		**(0.5** **μg/kg)**		**(2.5** **μg/kg)**		**(50** **μg/kg)**		**(250** **μg/kg)**	
		**Average**	**RSD**	**Average**	**RSD**	**Average**	**RSD**	**Average**	**RSD**
		**recovery(%)**	**(%)**	**recovery(%)**	**(%)**	**recovery(%)**	**(%)**	**recovery(%)**	**(%)**
*S*–naringenin	orange pulp	95.6	2.7	111.2	1.5	91.0	1.5	95.6	1.7
	orange peel	85.3	2.2	98.0	1.1	97.7	1.2	98.1	1.9
*R*-naringenin	orange pulp	93.3	1.7	110.0	3.0	98.0	1.2	101.1	3.8
	orange peel	87.2	3.6	110.3	1.2	90.9	0.9	101.6	1.2

The limit of detection (LODs) were calculated as three times the signal-to-noise ratio of the quantifier ion transition by the analyses of spiked sample containing naringenin at low concentration levels with five replicate extractions. The LOQs were defined as the lowest spiking level of each enantiomer on acceptable recovery. The LODs for both naringenin enantiomers were estimated to be 0.1 μg/kg in orange pulp and peel. The LOQs were established as being 0.5 μg/kg in orange pulp and peel based on five replicate extractions at lowest fortified level.

#### Application to Actual Samples

**Figure 6:**
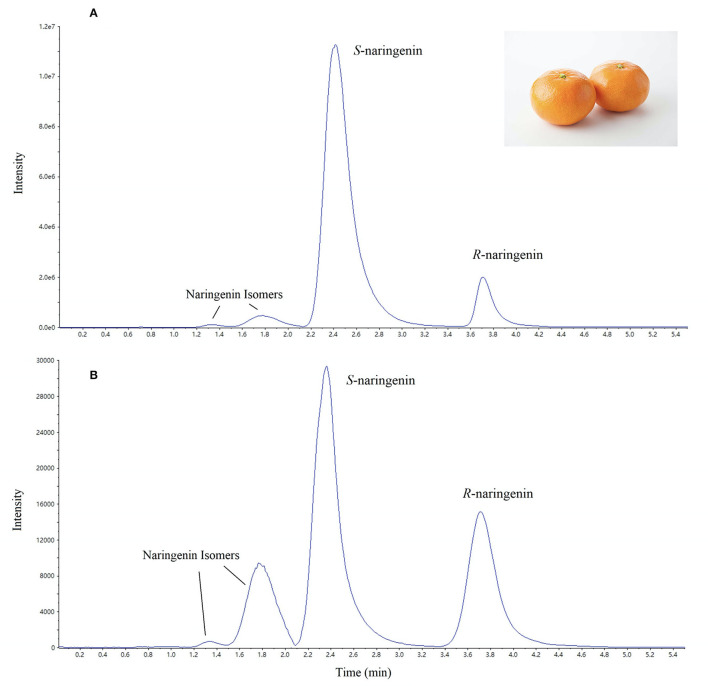
Typical mass spectra of real citrus samples. **(A)** citrus peel and **(B)** citrus pulp.

To further demonstrate the application and performance of the described method of naringenin and preliminarily explore the content characteristics of naringenin enantiomers in citrus tissues, different varieties of citrus samples were analyzed. Each sample was analyzed in quintuplicate. Typical spectra of naringenin enantiomers in citrus pulp and peel are shown in Figure 6. Enantiomeric excess (%) was calculated which showed excess of one enantiomer over the other in the naringenin enantiomer mixture. Enantiomeric excess = ([*S*]–[*R*]) / ([*S*]+[*R*]) × 100%, where [*S*] and [*R*] are *S*-naringenin and *R*-naringenin contents in orange samples, respectively. A positive value of enantiomeric excess indicates that the content of *S*-enantiomer was higher than that of *R*-enantiomer.

The contents of *S*- and *R*-naringenin in the pulp of 10 citrus cultivars involved in this study were 17.5–132.2 and 9.8–103.5 mg/kg·FW (Table 3). The content of *S*-naringenin was significantly higher than *R*-naringenin in the pulp of Hongmeiren, grapefruit, Zaojin navel orange, Sijiyou pomelo, and Zaoxiangyou pomelo, while the enantiomeric excesses were 24.5–27.3%. The negative value of enantiomeric excesses in Omishima navel orange and Seike navel orange pulp indicated that *R*-naringenin content was slightly higher than *S*-naringenin. The contents of naringenin enantiomers in the pulp of hybrid citrus and orange cultivars were significantly higher than that in pomelo.

**Table 3:** Application in determination of naringin enantiomers in different citrus pulp samples.

**Variety**	***S*** **-naringenin**		***R*** **-naringenin**		**Enantiomeric**
	**Content**	**RSD**	**Content**	**RSD**	**excess**
	**(mg/kg·FW)**	**(%)**	**(mg/kg·FW)**	**(%)**	**(%)**
Hongmeiren	132.2	1.9	65.7	0.4	33.6
Grapefruit	94.5	1.0	55.8	1.5	25.7
Zaojin navel orange	53.8	0.6	32.6	0.7	24.5
Midknight valencia orange	31.7	0.8	26.7	1.1	8.6
Blood orange	43.4	0.4	28.8	2.4	20.2
Fukumoto navel orange	98.2	0.4	66.6	0.4	19.2
Omishima navel orange	99.4	1.8	103.5	0.5	−2.0
Seike navel orange	70.3	0.7	72.1	2.3	−1.3
Sijiyou pomelo	30.0	0.8	13.7	0.8	37.3
Zaoxiangyou pomelo	17.5	0.9	9.8	3.2	28.2

The contents of *S*- and *R*-naringenin in the peel of eight citrus cultivars were 104.7–643.3 and 44.1–221.3 mg/kg·FW, with RSD of 0.7–5.0% and 0.2–4.7%, respectively (Table 4). The content of *S*-naringenin in the peel of eight citrus cultivars was significantly higher than that of *R*-naringenin. The enantiomeric excesses were 40.7–67.9%. Meanwhile, naringenin content in citrus peel was significantly higher than that in pulp, except Hongmeiren. The differences between *S*- and *R*-naringenin in the peel of Zaojin navel orange and blood orange were significantly higher than other varieties, and the *S*-naringenin content was 4.12 and 5.23 times of *R*-naringenin, respectively. These results indicated that the chiral isomers of naringenin showed differences in different citrus varieties and parts of fruits. Therefore, it is necessary to further analyze these phenomena *via* the synthesis and transformation mechanism of naringenin.

**Table 4:** Application in determination of naringin enantiomers in different citrus peel samples.

**Variety**	***S*** **-naringenin**		***R*** **-naringenin**		**Enantiomeric**
	**Content**	**RSD**	**Content**	**RSD**	**excess**
	**(mg/kg·FW)**	**(%)**	**(mg/kg·FW)**	**(%)**	**(%)**
Hongmeiren	104.7	3.6	44.1	3.3	40.7
Grapefruit	376.1	2.4	139.4	3.3	45.9
Zaojin navel orange	370.6	3.4	89.9	2.7	61.0
Midknight valencia orange	430.7	1.7	146.2	2.8	49.3
Blood orange	492.6	0.7	94.1	1.9	67.9
Fukumoto navel orange	294.5	2.6	118.4	1.1	42.6
Omishima navel orange	643.3	3.2	221.3	0.2	48.8
Seike navel orange	282.9	5.0	92.0	4.7	50.9

## Conclusion

This study used an enantioselective and UPLC-MS/MS method for analyzing naringenin enantiomers in orange. The absolute configuration of naringenin enantiomers was determined by the combination of experimental and predicted electronic circular dichroism spectra, and the first eluted enantiomer was confirmed as *S*-naringenin on a Chiralpak IG-3 column. LOQs of all naringenin enantiomers in citrus matrices did not exceed 0.5 μg/kg. Application of the proposed method to real sample analysis suggested its potential use in enantioselective determination of naringenin enantiomers in citrus.

The development of chiral UPLC-MS/MS method is beneficial to interdisciplinary research cooperation, pioneering the establishment of detection and analysis service platform, realizing achievement transformation and income generation, providing technical support for the breeding of characteristic citrus varieties and the development of high-quality cultivation techniques, and at the same time increasing the added value of fruit, which is beneficial to the expansion of functional consumption market and the extension of industrial chain of citrus.

## Data Availability Statement

The original contributions presented in the study are included in the article/supplementary material, further inquiries can be directed to the corresponding author/s.

## Author Contributions

ZY and SW conceived and designed the experiments and also wrote the original draft. ZY established the method based on UPLC-MS/MS for separation and determination of naringin enantiomer. SW, HZ, and ML analyzed the data. ZW calculated the theoretical ECD of naringenin enantiomer. XF and ML completed the collection and pretreatment of citrus samples from different varieties. All authors contributed to the article and approved the submitted version.

## Funding

This study was supported by grants from the National Natural Science Foundation of China (31801642), the Taizhou Science and Technology Plan Project (21nya23), and the Pioneer and Leading Goose R&D Program of Zhejiang (2022C02012).

## Conflict of Interest

The authors declare that the research was conducted in the absence of any commercial or financial relationships that could be construed as a potential conflict of interest.

## Publisher's Note

All claims expressed in this article are solely those of the authors and do not necessarily represent those of their affiliated organizations, or those of the publisher, the editors and the reviewers. Any product that may be evaluated in this article, or claim that may be made by its manufacturer, is not guaranteed or endorsed by the publisher.
